# Tumor Immune Microenvironment Related Gene-Based Model to Predict Prognosis and Response to Compounds in Ovarian Cancer

**DOI:** 10.3389/fonc.2021.807410

**Published:** 2021-12-13

**Authors:** Jiang Yang, Shasha Hong, Xiaoyi Zhang, Jingchun Liu, Ying Wang, Zhi Wang, Likun Gao, Li Hong

**Affiliations:** Department of Obstetrics and Gynaecology, Renmin Hospital of Wuhan University, Wuhan, China

**Keywords:** the tumor immune microenvironment, TIMErisk, prognosis, potential compound, ovarian cancer

## Abstract

**Background:**

The tumor immune microenvironment (TIME) has been recognized to be an imperative factor facilitating the acquisition of many cancer-related hallmarks and is a critical target for targeted biological therapy. This research intended to construct a risk score model premised on TIME-associated genes for prediction of survival and identification of potential drugs for ovarian cancer (OC) patients.

**Methods and Results:**

The stromal and immune scores were computed utilizing the ESTIMATE algorithm in OC patient samples from The Cancer Genome Atlas (TCGA) database. Weighted gene co-expression network and differentially expressed genes analyses were utilized to detect stromal-and immune-related genes. The Least Absolute Shrinkage and Selection Operator (LASSO)-Cox regression was utilized for additional gene selection. The genes that were selected were utilized as the input for a stepwise regression to construct a TIME-related risk score (TIMErisk), which was then validated in Gene Expression Omnibus (GEO) database. For the evaluation of the protein expression levels of TIME regulators, the Human Protein Atlas (HPA) dataset was utilized, and for their biological functions, the TIMER and CIBERSORT algorithm, immunoreactivity, and Immune Cell Abundance Identifier (ImmuCellAI) were used. Possible OC medications were forecasted utilizing the Genomics of Drug Sensitivity in Cancer (GDSC) database and connectivity map (CMap). TIMErisk was developed based on ALPK2, CPA3, PTGER3, CTHRC1, PLA2G2D, CXCL11, and ZNF683. High TIMErisk was recognized as a poor factor for survival in the GEO and TCGA databases; subgroup analysis with FIGO stage, grade, lymphatic and venous invasion, debulking, and tumor site also indicated similar results. Functional immune cells corresponded to more incisive immune reactions, including secretion of chemokines and interleukins, natural killer cell cytotoxicity, TNF signaling pathway, and infiltration of activated NK cells, eosinophils, and neutrophils in patients with low TIMErisk. Several small molecular medications which may enhance the prognosis of patients in the TIMErisk subgroup were identified. Lastly, an enhanced predictive performance nomogram was constructed by compounding TIMErisk with the FIGO stage and debulking.

**Conclusion:**

These findings may offer a valuable indicator for clinical stratification management and personalized therapeutic options for OC patients and may be a foundation for future mechanistic research of their association.

## Introduction

Globally, human ovarian cancer (OC) is associated with the highest lethality among the gynecological malignancies; the mortality accounts for approximately 5% of all female cancer-related deaths (1). Patients with epithelial OC (EOC), the main histological type, are commonly diagnosed at the late clinical stage due to the lack of symptoms at an early stage associated with the lack of an effectual diagnostic marker. While great progress in EOC treatment has been made due to the advances in surgery, chemotherapy, radiotherapy, immunotherapy, and targeted therapy, progression-free survival (PFS) and overall survival (OS) show modest improvements among patients. One of the primary explanations is the absence of effectual predictive markers for prognosis. Recently, an increasing body of evidence shows gene expression signature assays to estimate survival among tumor patients (2–4). Thus, the identification and development of new immune-related predictive markers which can predict patient prognosis more accurately and outline individualized treatment plans may have high clinical value.

**Figure 1:**
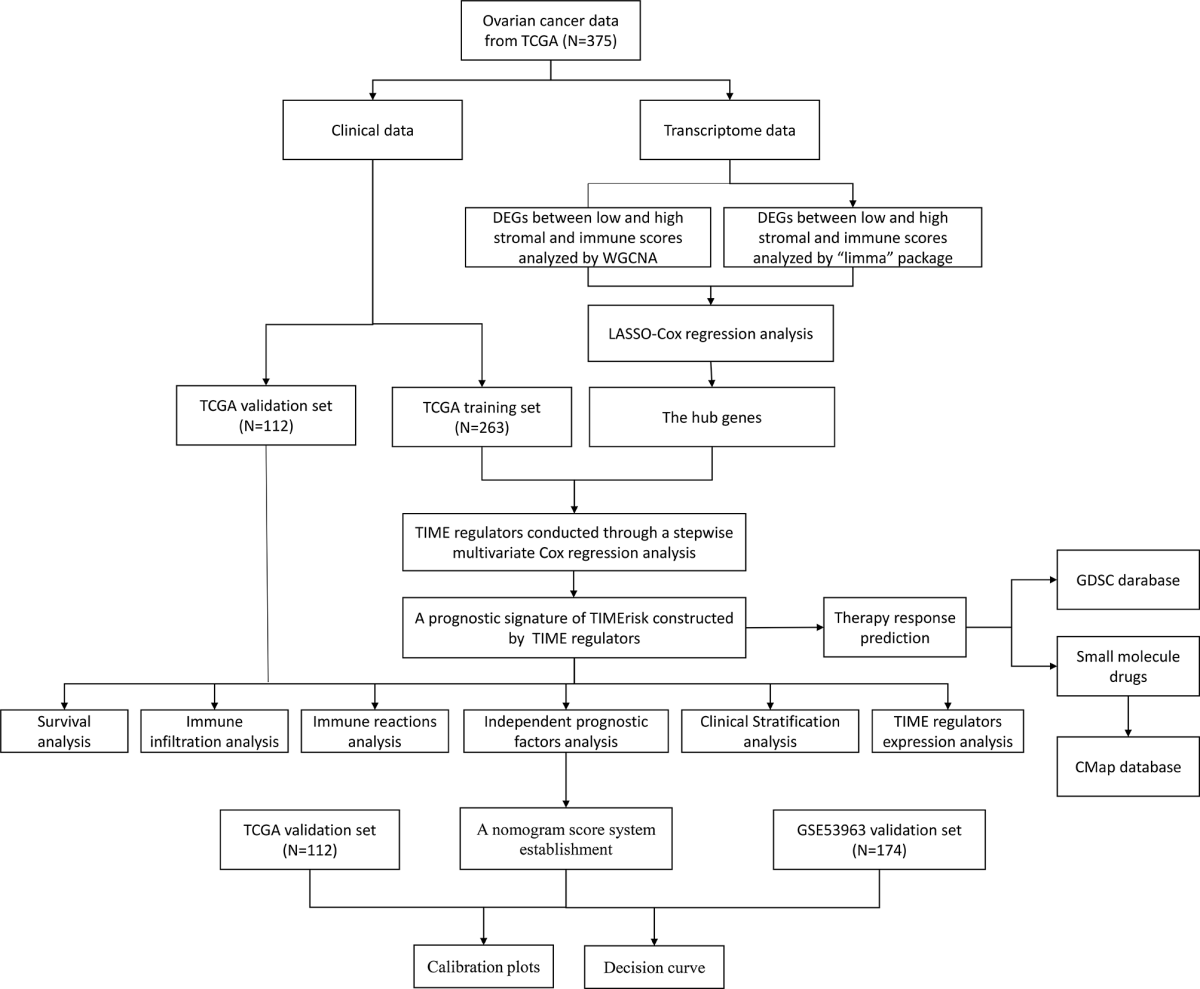
The workflow of the study.

The tumor immune microenvironment (TIME) has been known to be an important factor facilitating the acquisition of many cancer-related hallmarks. Successful peritoneal metastasis which entails the co-evolution of stromal and cancer cells has been recognized as the key cause for high risk of recurrence and mortality (5–7). In OC, several studies have shown the importance of TIME-related dysregulated and molecular signaling pathways as compared to the genomic factors; these can influence the patient’s survival probability, for example, there was a significant correlation between high lymphocyte infiltration and the survival time (8), the high immune scoring subtype with the upregulated tumor-infiltrating immune cells had a high BRCA1 mutation, high expression of immune checkpoints, and optimal survival prognosis (9, 10). In addition, mature DCs, M1 macrophages, natural killer (NK) cells, αβ T cells and γδ T cells can directly inhibit tumor growth or increase the susceptibility to checkpoint inhibitor therapies for OC (11, 12). Importantly, infiltration of CD4^+^ and CD8^+^ T cells into the tumor has been associated with improved overall and progression-free survival in OC patients (13).The TIME necessary for primary and metastatic outgrowth produces a target-rich niche for the development of auspicious anti-cancer agents. Although immune checkpoint inhibitors such as anti-PD-ligand 1 (anti-PD-L1), and anti-programmed death-1 (anti-PD-1) have been examined in phase III randomized controlled trials in OC patients (14), a perfect TIME related molecular biomarker to assess the susceptibility of drugs and prognosis of EOC, remains unknown. In this research, seven TIME-related genes were identified and a TIME-related risk model for prediction of survival and therapeutic responses in OC patients was constructed. The analysis workflow was shown in **Figure 1**.

## Materials and Methods

### Data Source and Evaluation of Stromal and Immune Scores

Gene expression data for patients with normal ovary and OC patients and their corresponding follow-up data were downloaded from GTEx (https://www.gtexportal.org/) and TCGA databases (https://portal.gdc.cancer.gov/). TCGA database was utilized to examine the possible TIME- and prognosis-related genes and premised on it the TIMErisk was then evaluated. An aggregate of 375 OC patients from TCGA, median age was 59 years (30 to 87), were randomly categorized into two cohorts in the ratio of 7:3 utilizing the “caret” package in R software; these were considered to be the training and testing cohorts, in that order. The training cohort consisting of 263 samples was utilized to detect the TIME regulators and build a prognostic model. The testing set with 112 samples was utilized to evaluate the model’s performance. GSE53963 and GSE140082 cohorts were accessed from the GEO dataset (https://www.ncbi.nlm.nih.gov/geo/) and utilized for independent external validation of the model. Stromal and immune scores were computed utilizing the “estimate” package in R software premised on the ESTIMATE algorithm (15).

### Analyses of Differentially Expressed Genes (DEG) and Weighted Gene Co-Expression Network (WGCNA) Based on RNA-Seq Data

The analysis of DEGs between low and high stromal and immune scores was carried out utilizing the “limma” package in R. The genes that had a false discovery rate (FDR) value (the adjusted P-value computed utilizing Benjamin–Hochberg method) <0.05 and | Log2 [Fold Change (FC)] | >2 were considered to be DEGs. WGCNA was utilized to detect the co-expressed gene modules strongly related to the stromal and immune scores. The gene modules of maximal |correlation coefficient were regarded as strong stromal and immune correlated modules. The intersection of stromal and immune correlated DEGs and strong stromal and immune-correlated gene modules was utilized as the input in the Least Absolute Shrinkage and Selection Operator (LASSO) regression analysis.

### LASSO Regularization and Construction of TIMErisk Model

LASSO is a descending dimension technique for regularization which could be utilized for examining biomarkers for survival analysis in combination with the Cox model (16). The stromal- and immune-related genes obtained from both the WGCNA and DEG analyses were employed as the input for the LASSO-Cox regression to detect the hub genes. Subsequently, a stepwise multivariate Cox proportional hazard regression analysis was conducted for these genes to obtain the ideal candidates and construct a prognostic model of TIMErisk using the TCGA training set. The genes with multivariate Cox P-value <0.05 were recognized as TIME regulators in the TIMErisk model. The model was calculated by the following formula: $\text{TIMErisk}=\Sigma_{i=1}^{n}\;coefi\times Xi$ , where “coefi” and “Xi” denote the coefficient and expression levels of each TIME regulator, in that order. Patients with EOC were categorized into low- and high-risk groups premised on the median TIMErisk score set as the risk threshold point. ROC (receiver operating characteristic) curve analysis was utilized to examine the distinctive performance of the TIMErisk model.

### Analysis of Tumor Immune Characteristics and Functional Enrichment for TIMErisk

The levels of infiltrating stromal and immune cells were computed utilizing the CIBERSORT algorithm (17). Single-sample gene-set enrichment analysis (ssGSEA) was utilized to estimate the activity of particular immune reactions, which in turn was utilized to delineate the enrichment score to represent the extent of absolute enrichment in the gene set for each of the samples within a specified data set (18). The gene sets for immune reactions were gotten from the ImmPort database (http://www.immport.org). The possible enriched biological functions associated with TIMErisk were detected utilizing the gene set enrichment analysis (GSEA) technique and annotated *via* Kyoto encyclopedia of genes and genomes (KEGG) and gene ontology (GO) datasets. In GSEA, the FDR value < 0.05 was judged to be a statistically significant enrichment.

### Construction and Validation of Nomogram Score System

To assess the independent prognostic significance, multivariate and univariate Cox regression analyses for TIMErisk and clinical variables were performed. Based on the independent prognostic significance a nomogram was built for the training set to forecast the survival of EOC patients. In the nomogram score system, each of the variables was allotted a point; total points were calculated by summation of points for each sample to predict survival. Using calibration plot, and decision curve analysis (DCA) the discriminating ability, and predictive performance of the nomogram score system were assessed. The nomogram was also employed in the testing set and the whole set to validate the results.

### Prediction of Chemotherapeutic Response and Small Molecule Drugs

The chemotherapeutic response in OC patients was evaluated utilizing a public dataset, Genomics of Drug Sensitivity in Cancer (https://www.cancerrxgene.org). Estimation of the drug response was done by computing the half-maximum inhibitory concentration (IC50). Possible small molecule drugs for UCEC were forecasted utilizing the Connectivity map (CMap, https://www.broadinstitute.org/connectivity-map-cmap) comprising of genome-wide transcriptional expression data from small molecule drugs; premised on the links between genes, drugs, and diseases *via* the variation in gene-expression profiles potential candidates were predicted. This was premised on DEGs between low- and high-risk cohorts with | log2fold change (FC) | > 2 and FDR < 0.05.

### Survival and Other Statistical Analyses

With regards to the categorical variables, Cox and KM regression analyses were utilized to compute the significant differences in the OS, which were then contrasted utilizing the log-rank test. With regards to continuous variables, Cox regression was utilized to compute the hazard ratio and significant differences in the OS. Stratified analysis was conducted to examine the survival differences among the subcategories, for instance, FIGO stage, age, grade, lymphatic and venous invasion, debulking, and tumor site. Spearman’s correlation analysis was conducted to determine the relationship between TIME regulators and immunocyte fractions and immune reaction activities. Two-tailed P-values were computed and P < 0.05 was judged to be statistically significant.

## Results

### Calculation of Stromal/Immune Scores and Their Relationship With Patients’ Clinical Characteristics

**Figure 2:**
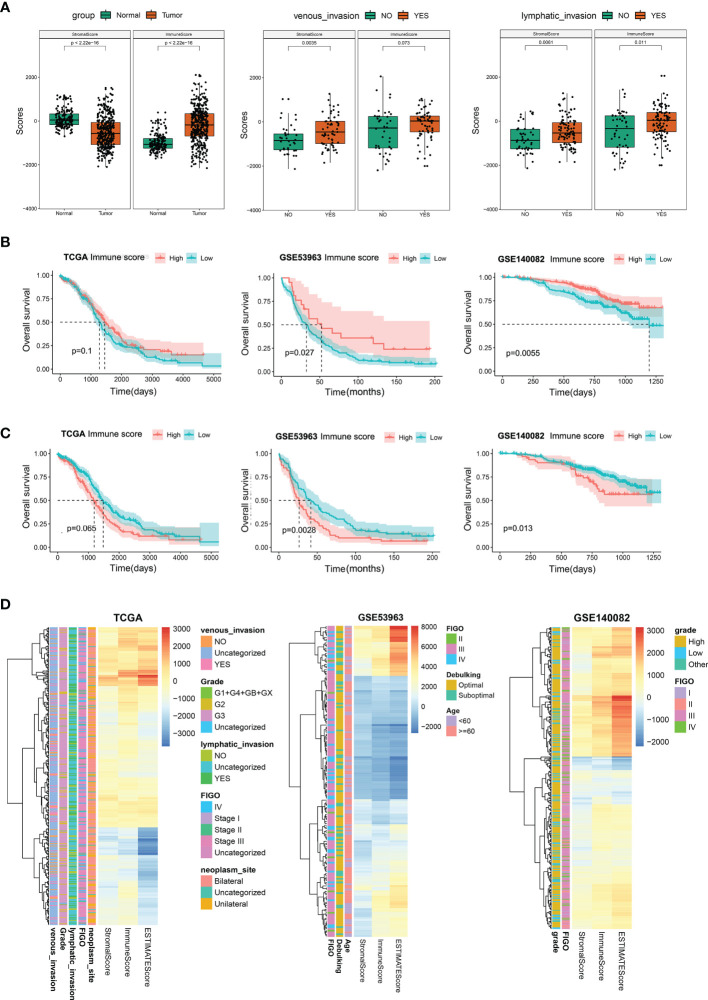
Stromal and immune scores and their relationship with patients’ clinical characteristics. **(A)** Differential analysis for the distribution of stromal and immune scores in tumor and normal tissues; in venous/lymphatic invasion and non- venous/lymphatic invasion in the tumor. **(B)** Survival analysis premised on the high- and low-immune scores in the TCGA, GSE53963, and GSE140082 databases. **(C)** Survival analysis premised on the high- and low-stromal scores in the TCGA, GSE53963, and GSE140082 databases. **(D)** Heatmap of immune, stromal, and ESTIMATE scores in TCGA, GSE53963, and GSE140082 databases.

To evaluate the role of immune infiltration in OC oncogenesis, an aggregate of 379 OC tissues from the TCGA dataset and 188 normal ovary tissues from the GTEx dataset were included in this research and the stromal and immune scores were computed. In OC patients, the range of the stromal span from −2098.5 to 1514.7 while the range of immune scores spans from −2153.5 to 2015.3. In normal tissues, the range of immune and stromal scores span from −1714.2 to 389.1 and −698.0 to 1171.5, in that order. As shown in **Figure 2A**, the stromal scores were considerably elevated in the normal as opposed to the tumor tissues, whereas the immune scores were higher in tumor tissues. This indicated that stromal and immune cells played opposite roles in oncogenesis. Furthermore, the stromal and immune scores in OC tissues were evaluated. We discovered that the stromal and immune scores were higher in patients with venous and lymphatic invasion as compared to those without tumor invasion (**Figure 2A**). In the FIGO stage, pathological grade, and different ages, the outcomes did not indicate any statistically significant differences between the cohorts, which indicated that stromal and immune cells could have an imperative function in tumor invasion and metastases (**Figures S1A–D**). KM analyses illustrated that the patients who had an elevated immune and reduced stromal scores exhibited better OS as opposed to those with reduced immune and elevated stromal scores (**Figures 2B, C**). Moreover, the stromal and immune scores of OC patients were determined in GSE53963 and GSE140082 cohorts, and the results of survival analysis were similar to those in the TCGA database (**Figures 2B, C**). The heatmap of immune and stromal scores for TCGA and GEO datasets are presented in **Figure 2D**. The above results suggested that the stromal and immune cells have a crucial influence on the tumorigenesis and prognosis in OC.

### Tumor Microenvironment Regulators Are Involved in OC

**Figure 3:**
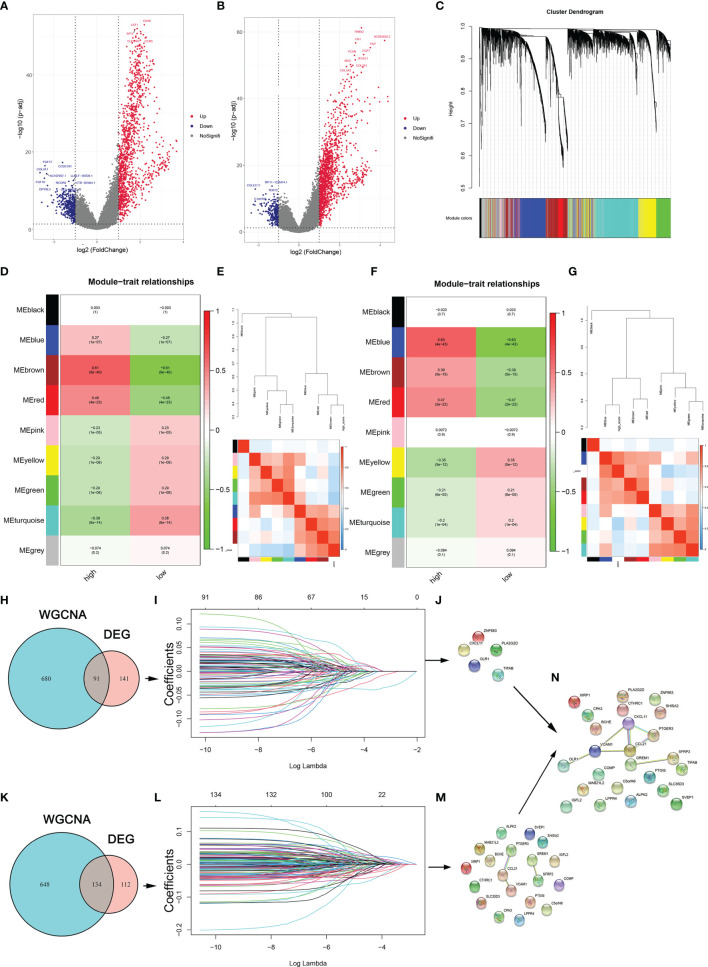
TME regulators are involved in the periodontitis process. **(A, B)** DEGs between the high- and low-stromal and immune score groups. **(C)** Clustering dendrograms of genes premised on dissimilarity topological overlap and module colors. **(D, F)** Scatter plots of immune and stromal genes significantly related to module membership. **(E, G)** Dendrogram of consensus module eigengenes and heatmap plot of the contiguities of modules for immune- and stroma-related genes. **(H, K)** Venn plot shows the number of intersecting immune- and stroma-related genes from DEG analysis and WGCNA. **(I, L)** LASSO analysis for choosing alternate genes associated with stromal and immune scores. **(J, M)** The significant genes are selected from immune-related genes and immune-related genes. **(N)** The genes are identified as the critical regulators in TIME.

To identify the genes involved in the regulation of tumor microenvironment, the limma package in R software was utilized and a WGCNA network was constructed to identify DEGs between the high- and low-risk cohorts premised on stromal and immune scores for OC in the TCGA database. The volcano plot depicted 232 DEGs in accordance with the immune scores and 246 DEGs in accordance with the stromal scores (**Figures 3A, B**). In WGCNA, the soft-thresholding power was adjusted at 3 with scale independence at adjusted to 0.9 to guarantee a scale-free network (**Figure S1E**). An aggregate of 24584 genes was assigned to 11 modules in the immune score groups, among which 771 genes belonged to the brown module. There were also 11 modules assigned in the stromal score groups; among them, 782 genes were assigned to the blue module (**Figures 3C–G**). Both the blue and brown modules were significantly linked to high stromal and immune scores, respectively (blue: correlation coefficient =0.93, P < 0:001; brown: correlation coefficient =0.92, P < 0:001; **Figures S1F, G**). The Venn plot was constructed and it displayed the number of DEGs as well as their intersection with strong TIME-correlated gene modules in immune and stromal score cohorts. These overlapping genes were further used as the input for LASSO-Cox regression analysis (**Figures 3H–L** and **Figures S1H, I**). Five genes (PLA2G2D, CXCL11, ZNF683, TIFAB, and OLR1) were selected from among the immune-related genes (**Figure 3J**); 19 genes (SFRP2, CCL21, GREM1, BCHE, IGFL2, COMP, SLC35D3, ALPK2, C5orf46, CPA3, SVEP1, PTGER3, MAB21L2, CTHRC1, XIRP1, LPPR4, PTGIS, and VCAM1) were selected from among the stromal-related genes (**Figure 3M**). To evaluated the prognostic precision of the gene set, the ROC curve analyses for OS was performed. The OS AUC values for the 19 risk signatures from the stromal score cohort and 5 risk signatures from the immune score cohort were 0.748 and 0.682, respectively (**Figures S2A, B**). Therefore, these 24 genes were identified as the critical regulators involved in TIME (**Figure 3N**).

### Establishment and Validation of Prognostic Signatures for Regulators of TIME

Further, the prognostic function of TIME regulators in OC patients was evaluated. An aggregate of 375 patients were categorized randomly into the TCGA training cohort (263 participants) and validation cohort (112 participants) in a ratio of 7:3 ratio. The basic features for the TCGA training and validation cohorts, such as FIGO stage, age, grade, lymphatic and venous invasion, days to the new tumor, and debulking, were not significantly different (all p > 0.05; **Table S1**).

**Figure 4:**
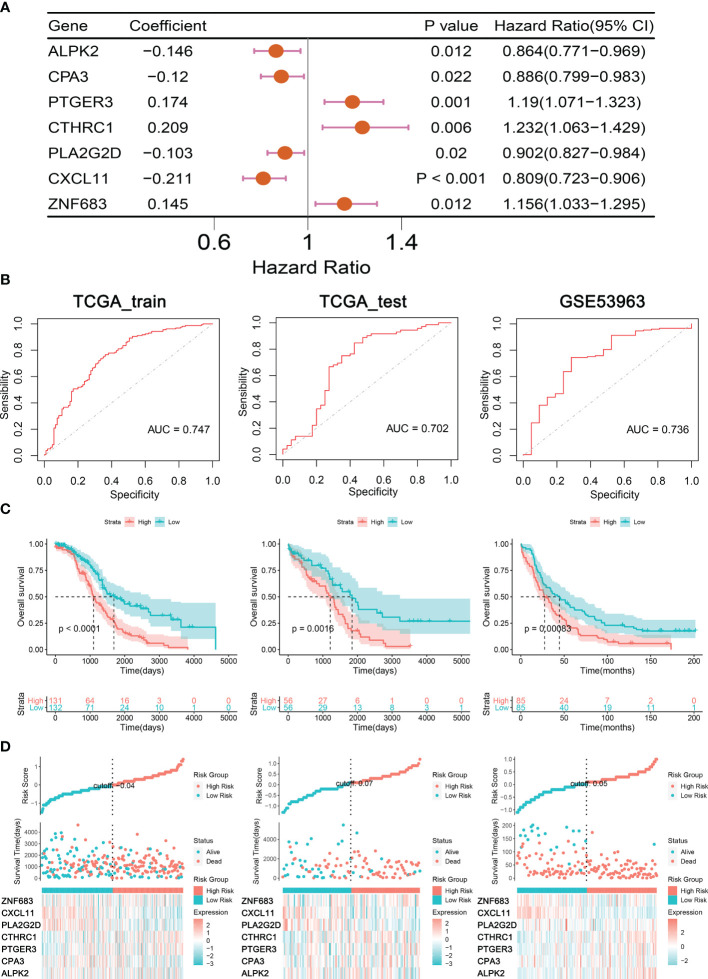
Construction and Validation of Prognostic Signatures for TIME regulators. **(A)** Forest plot presents the seven genes related to TIME. **(B)** ROC curves analysis of TIMErisk with OS in OC patients of TCGA training, testing, and GSE53963 cohorts. **(C)** Kaplan-Meier curve analysis of TIMErisk with OS in OC patients of TCGA training, testing, and GSE53963 cohorts. **(D)** A group of risk plots for TCGA training, testing, and GSE53963 cohorts, including the heatmap of TIME regulators’ expression and the distribution of patients’ survival status.

To accurately identify the significant genes for predicting clinical outcome in OC patients, multivariate Cox proportional hazard regression analysis was conducted in the TCGA training cohort; seven TME regulators, ALPK2, CPA3, PTGER3, CTHRC1, PLA2G2D, CXCL11, and ZNF683, were identified (**Figure 4A**). Subsequently, a prognostic signature built premised on the expression levels of these TME regulators and their coefficients in the multivariate Cox proportional hazard model were computed as follows: TIMErisk= 0.145×ZNF683 expression – 0.146× ALPK2 expression – 0.146× CPA3 expression +0.174 × PTGER3 expression +0.209 × CTHRC1 expression – 0.146× PLA2G2D expression – 0.146× CXCL11 expression. In the TIMErisk equation, three TIME regulators (ZNF683, PTGER3, and CTHRC1) had a positive coefficient which suggested that they were potential risk factors and the upregulation of their expression was linked to dismal prognosis. Four TIME regulators (ALPK2, CPA3, PLA2G2D, and CXCL11) with a negative coefficient were recognized to be protective factors that indicated an improved survival owing to the upregulation of their expression. Premised on the median TIMErisk score threshold of 0.83, patients whose scores were greater compared to the median in the training cluster were categorized as the high-risk cohort, while those whose scores were equivalent to or lower compared to the median were categorized as the low-risk cohort (**Table S2**). OC patients in the low TIMErisk cohort exhibited improved survival results contrasted with those in the high TIMErisk cohort (P < 0.001, log-rank test; **Figure 4C**). For the OS survival prediction of TIMErisk, the AUC of the ROC curves in the training set was 0.747 (**Figure 4B**).

To confirm the performance of the TIMErisk in predicting survival, we computed the TIMErisk scores for the testing cluster and the GSE53963 set; The outcomes indicated that their median TIMErisk scores were 0.941 and 0.017, respectively. Premised on the ROC curve and KM analyses (**Figure 4B**), patients in the low-risk cohort displayed prolonged survival as opposed to those in the high-risk cohort; AUC values were 0.702 and 0.736 in the testing and GSE53963 sets, respectively (**Figure 4C**). Furthermore, a set of risk plots for 3 databases, such as heatmap of TIME regulators’ expression and the spread of patients’ survival status were plotted. As depicted in **Figure 4D**, the expression levels of PTGER3 and CTHRC1 in the training, testing, and GSE53963 clusters increased as the TIMErisk scores increased, while the expression of CPA3, PLA2G2D, and CXCL11 reduced as the TIMErisk scores increased. Taken together, these results indicated that TIMErisk had a good efficacy for survival prediction.

### Association of TIME Regulators With EOC Clinical Characteristics

**Figure 5:**
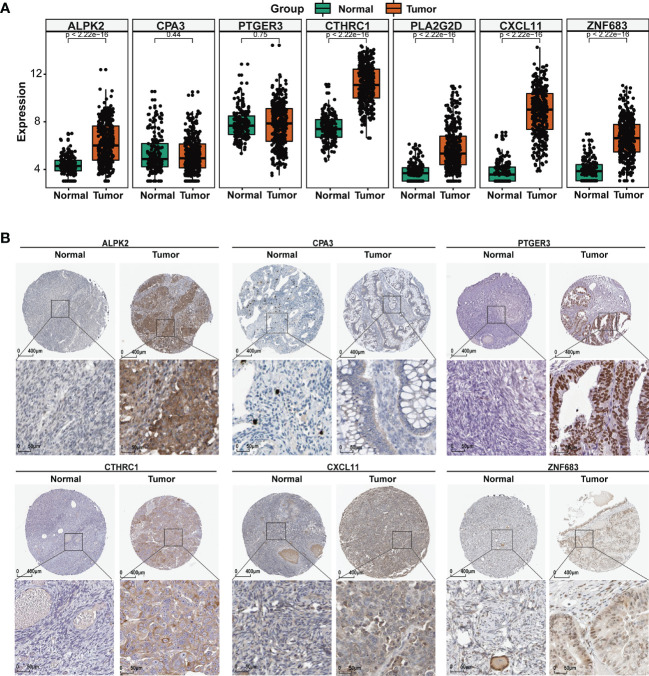
TME regulators are associated with EOC clinical characteristics. **(A)** Differential analysis of the mRNA expression of the seven TIME regulators between the normal ovary and OC tissues. **(B)** The protein expression of the seven TIME regulators in normal ovary and OC tissues using the HPA database.

To identify the role of the seven TIME regulators in OC tumorigenesis, we analyzed their expression in normal ovary and OC tissues from the GTEx and TCGA databases. As shown in **Figure 5A**, all TIME regulators except CPA3 and PTGER3 were overexpressed in the OC. Furthermore, the protein expression of TIME regulators was analyzed using the Human Protein Atlas (HPA) database; six of the seven TIME regulators (ALPK2, CPA3, PTGER3, CTHRC1, CXCL11, and ZNF683) were analyzed and all except CPA3 had enhanced expression in OC (**Figure 5B**). Interesting, it appears that PTGER3 expression is non-significant at mRNA level, while the protein expression appears significant in the tumor sections, which may be caused by the regulation of epigenetics. So, the results above suggested that the TIME regulators might have a vital function in the oncogenesis of OC.

To elucidate the underlying role of the TIME regulators in the development of OC, the mRNA expression levels for different clinical characteristics were analyzed in the TCGA database. As shown in **Figure S3A**, PTGER3 and PLA2G2D were overexpressed and thus, contributed to lymphatic and venous invasion; the aberrant expression of ALPK2, CPA, PTGER3, and PLA2G2D were closely associated with FIGO stage; PLA2G2D was related to the pathological grade, and lower expression of PLA2G2D and CXCL11 was found in patients with bilateral as compared to unilateral ovarian cancer; high expression of PTGER3 and CTHRC1 increased the difficulty in debulking, and the expression of seven TME regulators did not show any significant differences with age Furthermore, the correlation between the expression of TIME regulators and OS in OC patients was determined. As depicted in **Figure S3B**, patients that had an elevated expression of PLA2G2D and CXCL11 displayed longer survival, while the expression of the other five TIME regulators had no significant association with OS. The above results indicated that OC oncogenesis and development involved the participation of multiple genes; studies based on single genes may lead to bias in results. Therefore, using the significant gene set is relevant to the evaluation of the clinical prognosis of OC.

### Association of TIMErisk With EOC Immune Signature

**Figure 6:**
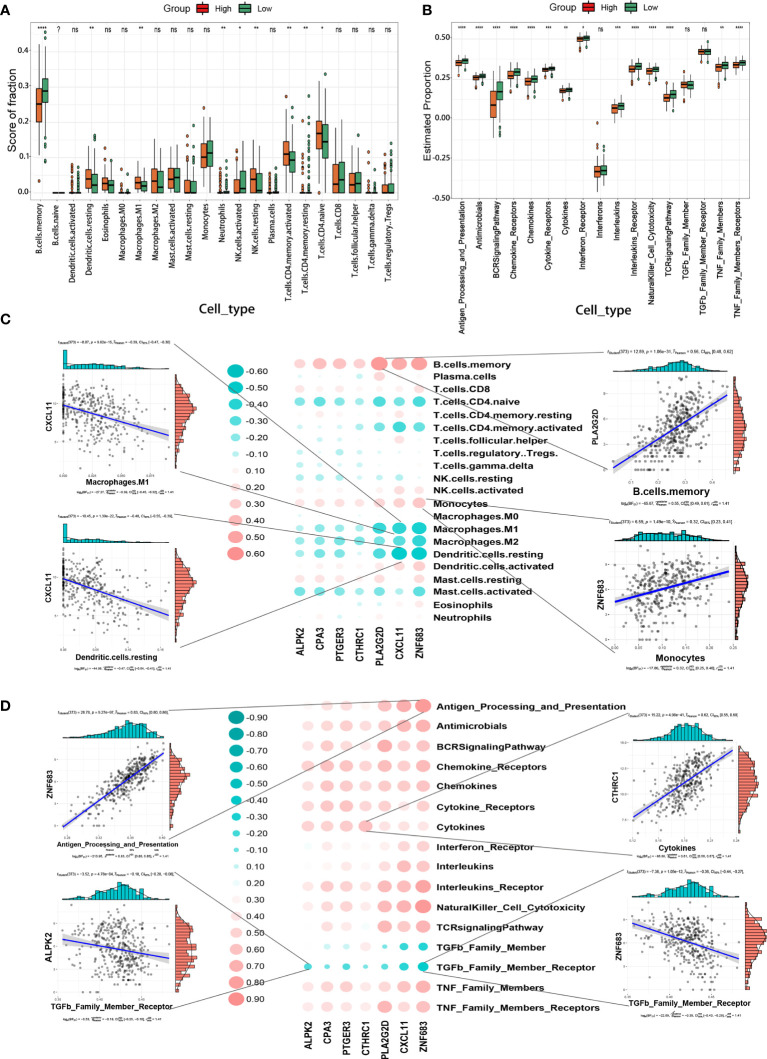
TIMErisk is associated with EOC immune signature. **(A)** The differences in abundances of each immune microenvironment infiltrating immunocyte for TIMErisk subgroup in TCGA training cohort. **(B)** The activity differences for each immune reaction gene-set of TIMErisk subgroup in TCGA training cohort. **(C)** Dot-plot demonstrates the correlation between each dysregulated immune microenvironment infiltration cell type and TIME regulators in the TCGA training cohort. **(D)** Dot-plot demonstrates the correlation between dysregulated immune reaction gene-set and the TIME regulators in TCGA training cohort. Data are presented as mean ± SD, ns p > 0.5. *p < 0.5; **p < 0.01; ***p < 0.001; ****p < 0.0001.

To assess the differences in features of the immune microenvironment between the high- and low-TIMErisk cohorts, infiltrating immunocytes and immune reaction gene-sets were evaluated. Infiltration of several immunocytes differed between the two groups (**Figure 6A**). High-TIMErisk group had higher levels of CD4 memory-activated T cells, CD4 naive T cells, M1 macrophages, resting NK cells, and resting dendritic cells; memory-activated B cells, eosinophils, activated NK cells, and neutrophils were enriched in low-TIMErisk groups. This suggested that the high TIMErisk group had a relatively different infiltration of immunocytes. Active immune reactions were higher in the low TIMErisk group. For example, antigen processing and presentation, chemokines, natural killer cell cytotoxicity, TNF family members, interleukins, and BCR signaling pathways were active in the low TIMErisk group (**Figure 6B**). These results suggested that the low TIMErisk group mediated an active immune response, while those in the high TIMErisk group involved active immunosuppression of OC.

Furthermore, to investigate the biological behavior of TIME regulators in the immune microenvironment, we analyzed the correlation of TIME regulators with immune reaction gene-sets and infiltrating immunocytes. As shown in **Figure 6C**, the expression of TIME regulators, especially PLA2G2D, CXCL11, and ZNF683, exhibited a positive correlation with memory B cells and monocytes, and a negative correlation with M2 macrophages, CD4 memory activated T cells, CD4 naive T cells, M1 macrophages, and activated mast cells. Remarkably, the expression of TIME regulators exhibited a negative relationship with the immune reactions mediated by the members of the TNF family and positively correlated with most other immune reactions, including antigen processing and presentation, chemokines, natural killer cell cytotoxicity, and BCR signaling pathways (**Figure 6D**). This indicated that TIME regulators, in particular, the aberrant expression of PLA2G2D, CXCL11, and ZNF683 may play different roles in the process of immune infiltration and could lead to strong immune reactions in OC,

### Assessment of Independent Prognostic Significance of TIMErisk and Clinical Stratification

**Figure 7:**
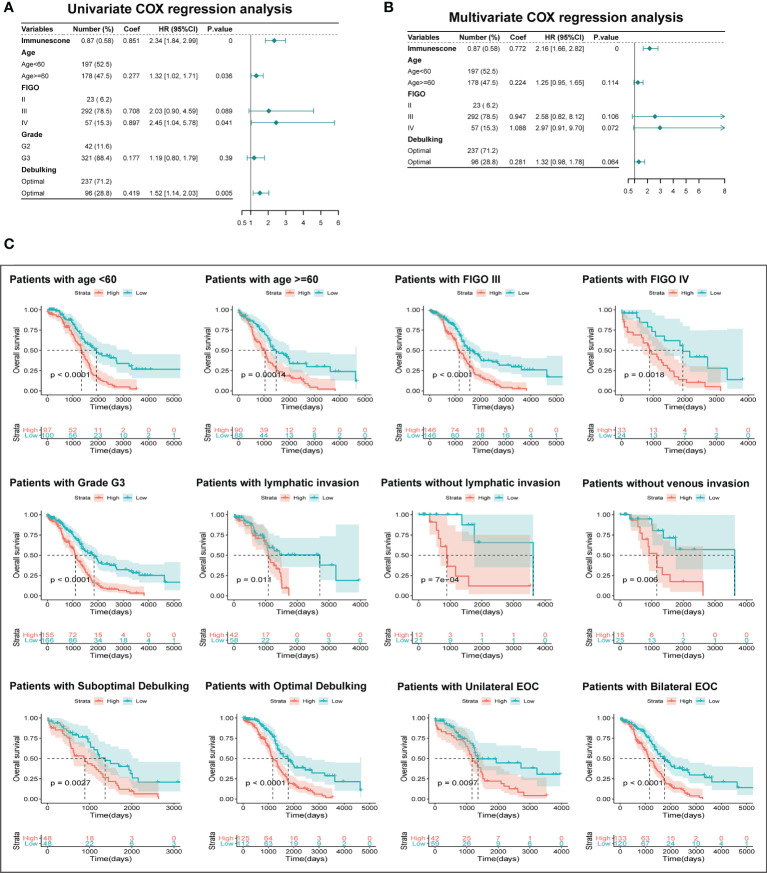
Independent Prognostic Significance of TIMErisk and Clinical Stratification Analysis. **(A)** Univariate Cox regression analysis of the TIMErisk and clinical characteristics for the independent prognostic significance in the TCGA cohort. **(B)** Multivariate Cox regression analysis of the TIMErisk and clinical characteristics for the independent prognostic significance in the TCGA cohort. **(C)** Kaplan–Meier survival curves of patients in the low- and high-risk groups within clinically stratified subcategories.

To examine if TIMErisk was an independent prognostic factor of clinicopathological characteristics, we conducted multivariate and univariate Cox regression analyses utilizing the TCGA datasets for variables such as Immunescore, age, FIGO stage, grade, and debulking. The outcomes of univariate Cox regression illustrated that Immunescore, age, FIGO stage, and debulking had a significant correlation with the patients’ OS (P <0.05). Through multivariate Cox regression analysis, Immunescore, FIGO stage, and debulking were identified as the prognostic significances (P < 0.1, **Figures 7A, B**). Furthermore, clinical stratification for the analysis of TIMErisk prognostic performance was conducted in the TCGA database after adjusting for other clinical features such as age, FIGO stage, grade G3, lymphatic invasion, debulking, and tumor site. The outcomes illustrated that patients in the low-risk cohorts had improved survival outcomes as opposed to those in the high-risk cohorts among all clinically stratified subcategories (P < 0.05, **Figure 7C**). To surmise, these findings indicated that the prognostic value of TIMErisk in OC patients was independent of other clinicopathological characteristics.

### Construction and Validation of Nomogram for Survival Prediction of EOC Patients

**Figure 8:**
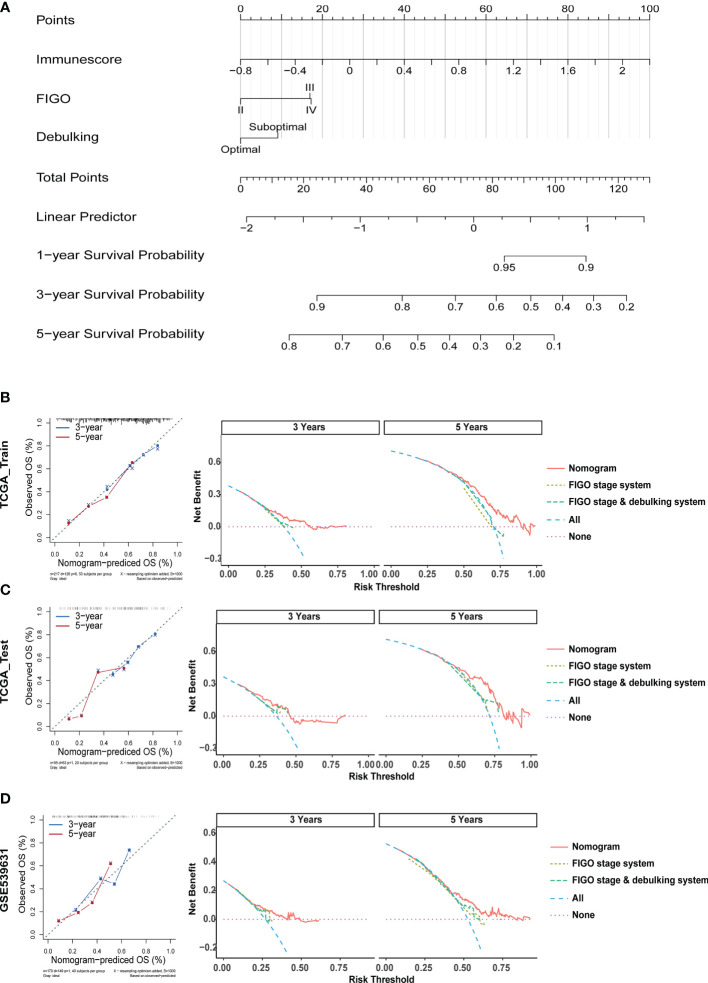
Construction and Validation of a Nomogram for Survival Prediction of EOC Patients. **(A)** The nomogram was constructed in the training cohort for forecasting 1-, 3-, and 5-year survival of patients. **(B)** Calibration plots and decision curves for 3-, and 5-year survival prediction premised on the nomogram in the TCGA training cohort. **(C)** Calibration plots and decision curves for 3-, and 5-year survival prediction premised on the nomogram in the TCGA test cohort. **(D)** Calibration plots and decision curves for 3-, and 5-year survival prediction premised on the nomogram in the GSE53963 cohort.

To enhance the applicability of the model in clinical practice, we constructed a statistical nomogram model in the training cohort by combining the prognostic significances (Immunescore, FIGO stage, and debulking) using “rms” and “survival” packages in R (**Figure 8A**). The calibration plots depicted in **Figures 8B–D** displayed that the nomogram had better performance as opposed to that of an ideal model based on the training, test, and GSE53963 cohorts. Similarly, according to the decision curve (**Figures 8B–D**), the nomogram also showed a higher net benefit and better predictive accuracy than the FIGO stage system and FIGO stage & debulking system. Thus, these outcomes illustrated that the nomogram had improved prediction ability.

### New Therapeutic Regimens for OC

**Figure 9:**
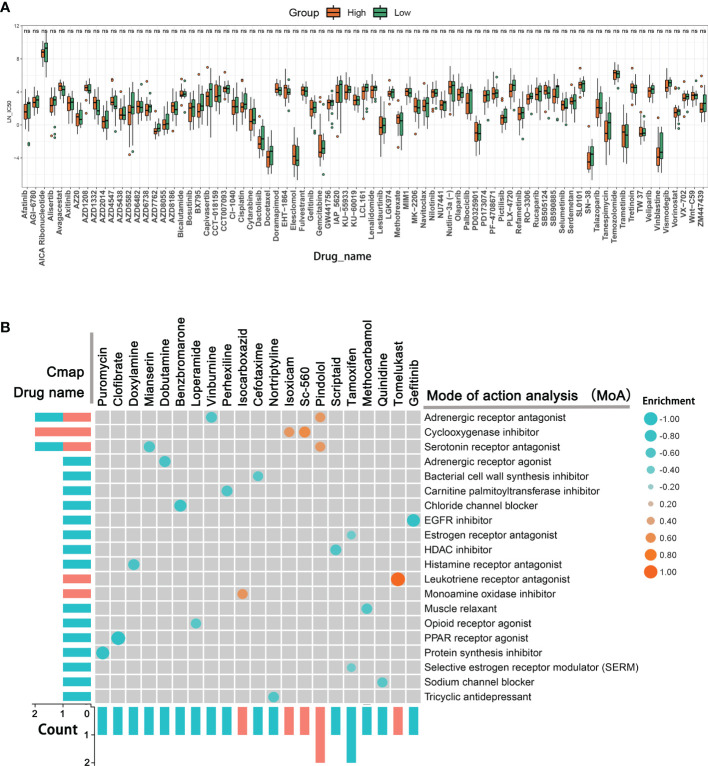
New therapeutic regimens for OC. **(A)** The IC50 differences for 79 drugs between high- and low-TIMErisk clusters in TCGA database. **(B)** Novel candidate drugs targeting the TIME-related signature identified by CMap database analysis.

To further identify the relevant drugs for high- and low-TIMErisk patients, the GDSC database consisting of cancer cell responses to various drugs was downloaded. the IC50 of 79 drugs in 33 ovarian cancer cells was estimated. Surprisingly, we found that high- and low-TIMErisk clusters exhibited similar cytotoxicity to all drugs, including the chemotherapeutic drugs cisplatin, docetaxel, gefitinib, and the bio-targeted drug olaparib (**Figure 9A**). To explore new therapeutic regimens for OC, the DEGs between the high- and low- TIMErisk cohorts were identified; GO enrichment analysis illustrated that the DEGs primarily participated in the regulation of humoral immune response, immune response activating signal transduction, and adaptive immune response premised on somatic recombination of immune receptors constructed from immunoglobulin superfamily domains (**Figure S4A** and **Table S3**). In addition, GSVA enrichment analysis was utilized to assess the activation status of KEGG pathways. As opposed to the low TIMErisk cohort, patients in the elevated TIMErisk cohort exhibited a greater pathway enrichment in primary immunodeficiency, chemokine signaling, and natural killer cell-mediated cytotoxicity (**Figure S4B** and **Table S4**). Furthermore, we sought to examine potential drugs targeting the pathways of the DEGs between low- and high TIMErisk cohorts in the CMap dataset. The CMAP mode-of-action (MoA) analysis of 21 compounds illustrated that 20 MoAs were shared between these drugs (**Figure 9B** and **Table S5**). CMap database analyses showed that two drugs (vinburnine and pindolol) shared the MoA with the adrenergic receptor antagonist, Isoxicam and Sc-560 shared the MoA with cyclooxygenase inhibitor, and mianserin and pindolol shared their MoA with serotonin receptor antagonist. Patients with low TIMErisk showed greater sensitivity for protein synthesis inhibitor (puromycin) and PPAR receptor agonist (clofibrate); cyclooxygenase inhibitor (Isoxicam and Sc-560) and leukotriene receptor antagonist (tomelukast) may offer possible advantages to patients in the high TIMErisk cohort. Thus, drugs targeting the TIMErisk signature were identified and these may provide therapeutic targets for future experiments.

## Discussion

TIME has an intensive interplay between tumor, stromal, and immune cells, and these interactions are critical for tumorigenesis and cell proliferation. Aberrant infiltration of immune and stromal cells in human tumors not only fails to restrain tumor growth but also promotes tumor escape from the host and thereby influences the patient’s prognosis (19, 20). Low-level infiltration of cytotoxic immune cells could contribute to the escape of the tumor cells from immune attack. It can increase tumor-infiltrating T cells and clear tumor cells efficiently due to their immune-active nature (21). The stroma can regulate tumor immune phenotypes, including cancer-related fibroblasts (CAFs) which suppress the antitumoral immune response due to the entry of T cells (22). OC is capable of create a highly complex and heterogenous ecosystem where anti-tumor immune cells may be hijacked to evade human immune attack (23). In this research, the stromal and immune scores for normal ovary and OC tissues were computed utilizing the ESTIMATE algorithm. We found that the normal tissues had increased stromal and reduced immune scores as opposed to the OC tissues, which suggested that the remodeled ECM and alternate immune cells could have a crucial function in OC tumorigenesis. Previous studies show that high stromal and immune scores are linked to dismal survival in gastric cancer (24), and better survival in lung adenocarcinoma (3). However, we discovered that an elevated immune score was a significant factor for dismal prognosis and an elevated stromal score was linked to good OS in OC patients. These were also validated in the GSE53963 and GSE140082 datasets. Together, this indicated that the prognostic value of stromal and immune scores may exhibit distinct values in solid tumors. Interestingly, we discovered that the elevated stromal and immune scores were correlated with the venous and lymphatic invasion, unlike previous findings where a low stromal score was a factor for dismal prognosis in OC. Venous and lymphatic invasion are independent high-risk risk factors for poor prognosis (25, 26). This suggested that the stromal and immune cells had distinct roles at different stages of OC, and thus, a more detailed study is necessary to elucidate the TIME mechanisms.

Because of the poor prognosis and high recurrence in OC, clinically useful molecular prognostic biomarkers and intensification of individualized therapy are needed for high-risk patients. Therefore, we constructed a TIMErisk signature premised on immune and stromal scores which could accurately predict OC patient prognosis using only seven genes. The genes participating in the TIMErisk model embodied a negative or positive correlation with OS. The overexpression of PTGER3, ZNF683, and CTHRC1 could increase the TIMErisk score and lead to poor prognosis, whereas high expression of ALPK2, CPA3, PLA2G2D, and CXCL11 decreased TIMErisk score and contributed to better survival. The prostaglandin E2 (PGE2) receptor, PTGER3, exerts multiple effects including invasion, epithelial cell growth, and immune regulation (27). Our findings were similar to previous results which show that abnormal PTGER3 expression is linked to the biological hallmarks of malignancies exhibiting negative clinical prognosis (27–29). The elevated PTGER3 expression was correlated with dismal survival and high-risk factors of stage, lymphatic and venous invasion, and suboptimal debulking in OC patients. ZNF683, originally Hobit for Homolog of Blimp-1 in T cells discovered in 2010, is nearly entirely expressed in effector T cells (30). A few researchers have investigated its role in tumorigenesis and tumor progression. Although ZNF683 expression was exhibited no significant relationship with the prognosis of EOC, we found overexpression of ZNF683 in the patients with lymphatic invasion, late-stage, and high grade. This suggested that ZNF683 could target the TIME signaling cascade. Increasing evidence shows the important function of ZNF683 in long-lived and quiescent effector-type CD8+ T cells and differentiation of the human NK-cell lineage (31, 32). PLA2G2D is an immune regulator that participates in the transformation of lipid balance to an anti-inflammation status and can either have a beneficial or detrimental function, contingent on the inflammation and tumor context (33, 34). CXCL11 exhibits multiple effects such as modulation of angiostatic effects, cell adhesion, regulation of cell proliferation and self-renewal, and chemotactic migration. It is a biomarker for predicting prognosis and targeted therapeutic effects (35, 36). Similar to the above studies, PLA2G2D and CXCL11 which were overexpressed in the patients at an early stage, correlated with better survival. ALPK2 is essential in cancer as it regulates the cell cycle and DNA repair genes (37). CPA3 is involved in the transactivation of the p21^WAF1/CIP1^ gene and histone deacetylase (38). CTHRC1 modulates macrophage polarization to M2 phenotypes by directly binding to the TGF-β receptor II and TGF-β receptor III and activating TGF-β signaling (39). Aberrant expression of ALPK2 and CTHRC1 are independent high-risk factors for poor survival in multiple tumors (40, 41). In this study, ALPK2, CPA3, and CTHRC1 were overexpressed in the patients at a late stage and associated with the debulking difficulty. Therefore, tumorigenesis and development of OC involved the participation of multiple TIME genes and the TIMErisk signature was had better relevance for the evaluation of the clinical prognosis of OC.

Interestingly, resting or naïve immune cells such as CD4 naive T cells, resting dendritic cells and resting NK cells were elevated in the patients with higher TIMErisk, whereas a large number of functional immune cells, including activated NK cells, eosinophils, and neutrophils, corresponding to more incisive immune reactions, including antigen processing and presentation, chemokines, interleukins, natural killer cell cytotoxicity, and BCR and TNF signaling pathways showed higher infiltration in the low TIMErisk patients. The findings were consistent with the conclusion that immunosuppression was the primary cause for the greater risk of malignant progression and death. Based on the above results, we speculated that the predictive model based on TIMErisk may be a reliable biomarker for cancer therapy.

Fortunately, we identified 21 compounds with 20 MoAs, including adrenergic receptor antagonists, a cyclooxygenase inhibitor, and a serotonin receptor antagonist. The PPAR receptor agonist (clofibrate) and chloride channel blocker (benzbromarone), in particular, could offer possible advantages for high-risk cohort patients. Cyclooxygenase inhibitor (Sc-560) and leukotriene receptor antagonist (tomelukast) showed greater sensitivity for patients with low TIMErisk. Clofibrate is widely used in the treatment of tumors including pancreatic cancer (42), colon cancer (43), and acute myeloid leukemia (44). For approximately 30 years, benzbromarone has been used therapeutically for chronic gout and possesses cytotoxicity for hepatocellular carcinoma, which could be suppressed by CYP3A inhibitors, Nrf2 activator, and GSH precursor (45). Wei et al (46) show that SC-560 can exert significant cytotoxicity and cisplatin or taxol supplementation in human ovarian cancer xenografts enhances the inhibition effect on angiogenesis as compared to cisplatin or taxol alone. Tomelukast is commonly used to treat asthma and related respiratory disorders (47). Although its effect on the tumor is unknown, tomelukast could target the PPARα and PPARγ to regulate carbohydrate and lipid metabolism, and exert an anti-inflammatory action (48). The aforementioned medications are untraditional anti-tumor compounds and the existing evidence about their influence on tumors particularly in OC is limited. Nevertheless, among patients in the various TIMErisk subcategories, all medications with potential advantages ought to be evaluated. In patients who might not gain any improvement from the traditional medicine alone, adjuvants can be administered.

Currently, FIGO staging is the most extensively utilized technique to examine the malignancy potential as well as the disease progression in OC. However, this technique has its drawbacks since it is mainly contingent on the distant metastasis, lymph node invasion, size, and location of the tumor. It does not consider the heterogeneity of tumor, age, and other clinical characteristics. Therefore, it is costly to construct an integrated prognostic model consisting of clinical properties and high-risk gene signatures. Our results showed that FIGO stage, debulking, and TIMErisk of the analyzed EOC samples in the TCGA training cluster were independent high-risk factors associated with poor survival. In addition, the nomogram based on the prognostic signature showed a higher net benefit and better predictive accuracy than the FIGO stage system and FIGO stage & debulking system. These results may guide the individualized treatment for OC patients.

While our model was valuable in examining prognosis and carrying out therapies for OC patients, it ought to be prospectively confirmed by large-sample clinical studies. The context of TIME, such as remodeling stroma, the status of immune cells infiltration, immunoreaction, and the molecular mechanisms mediated by TIMErisk was explored through bioinformatic analysis only. Thus, additional experimental validations are required to corroborate these findings. Additionally, we have identified several potential drug compounds for TIMErisk subgroups. Unfortunately, there is a lack of available research to confirm their efficacy in ovarian cancer. Further investigations are needed to contrast the TIMErisk scores with present biomarkers and examine the relationship between TIMErisk and the potential drugs in OC patients.

## Conclusion

To summarize, our research detected a risk prognostic signature encompassing 7 genes related to TIME. The TIMErisk can forecast the OS of OC patients and indicated the situation of stroma remodeling and immune response. Candidate drugs for TIMErisk subgroups were identified. Lastly, an enhanced predictive performance nomogram by compounding TIMErisk with the FIGO stage and debulking was constructed. Ultimately, these findings could offer a valuable indicator for clinical stratification management and personalized therapeutic options for OC patients and may be a foundation for forthcoming mechanistic research projects of their association.

## Data Availability Statement

The original contributions presented in the study are included in the article/**Supplementary Material**. Further inquiries can be directed to the corresponding author.

## Author Contributions

JY, SH, and LH were responsible for the design, analysis, and draft of the research. JL, YW, and XZ plotted all figures in this manuscript. ZW and LG helped in data analysis. All authors approved the final version of this manuscript and agreed to be accountable for all aspects of the work.

## Funding

This research is sponsored by Hubei Province’s Outstanding Medical Academic Leader Programme and the Fundamental Research Funds for the Central Universities (No. 2042021kf0125).

## Conflict of Interest

The authors declare that the research was conducted in the absence of any commercial or financial relationships that could be construed as a potential conflict of interest.

## Publisher’s Note

All claims expressed in this article are solely those of the authors and do not necessarily represent those of their affiliated organizations, or those of the publisher, the editors and the reviewers. Any product that may be evaluated in this article, or claim that may be made by its manufacturer, is not guaranteed or endorsed by the publisher.

## Supplementary Material

Supplementary Table S1 The basic features for the TCGA training and validation cohorts.

Supplementary Table S2 The information of high-TIMErisk and low-TIMErisk cohorts in the training and testing clusters.

Supplementary Table S3 GO enrichment analysis for the DEGs between high-TIMErisk and low-TIMErisk cohorts.

Supplementary Table S4 KEGG enrichment analysis for the DEGs between high-TIMErisk and low-TIMErisk cohorts.

Supplementary Table S5 The CMAP mode-of-action (MoA) analysis of 21 compounds.

Supplementary Figure S1
**(A-D)** Differential analysis for the distribution of stromal and immune scores among FIGO stages; in pathological grade; in different ages and tumor site. **(E)** A scale-free network was constructed when the soft-thresholding power was set at 3. **(F, G)** Scatter plots of blue and brown modules are represented. **(H, I)** The distribution plot of the partial likelihood deviation of the LASSO coefficient in immune and stromal score cohorts.

Supplementary Figure S2
**(A, B)** ROC curves analysis of 19 risk signatures from the stromal score cohort and 5 risk signatures from the immune score cohort. **(C)** Differential analysis for the distribution of risk scores in different ages; among FIGO stages; in pathological grade; in days to new tumor; in debulking, in lymphatic and venous invasion; and in tumor sites.

Supplementary Figure S3
**(A)** Differential analysis for the expression of seven TIME regulators in different ages; in debulking; in lymphatic and venous invasion; among FIGO stages; and in pathological grade. **(B)** Kaplan-Meier curve analysis of seven TIME regulators with OS in OC patients of TCGA cohorts.

Supplementary Figure S4
**(A)** The differences of GO enrichment analysis between high TIMErisk and low TIMErisk cohorts. **(B)** The differences of KEGG pathway enrichment score between high TIMErisk and low TIMErisk cohorts.
