# Supplementary figures and images for: Tumor Immune Microenvironment Related Gene-Based Model to Predict Prognosis and Response to Compounds in Ovarian Cancer
Source: Front Oncol. 2021 Dec 13;11:807410. doi: 10.3389/fonc.2021.807410 (PMC8710702; doi:10.3389/fonc.2021.807410)

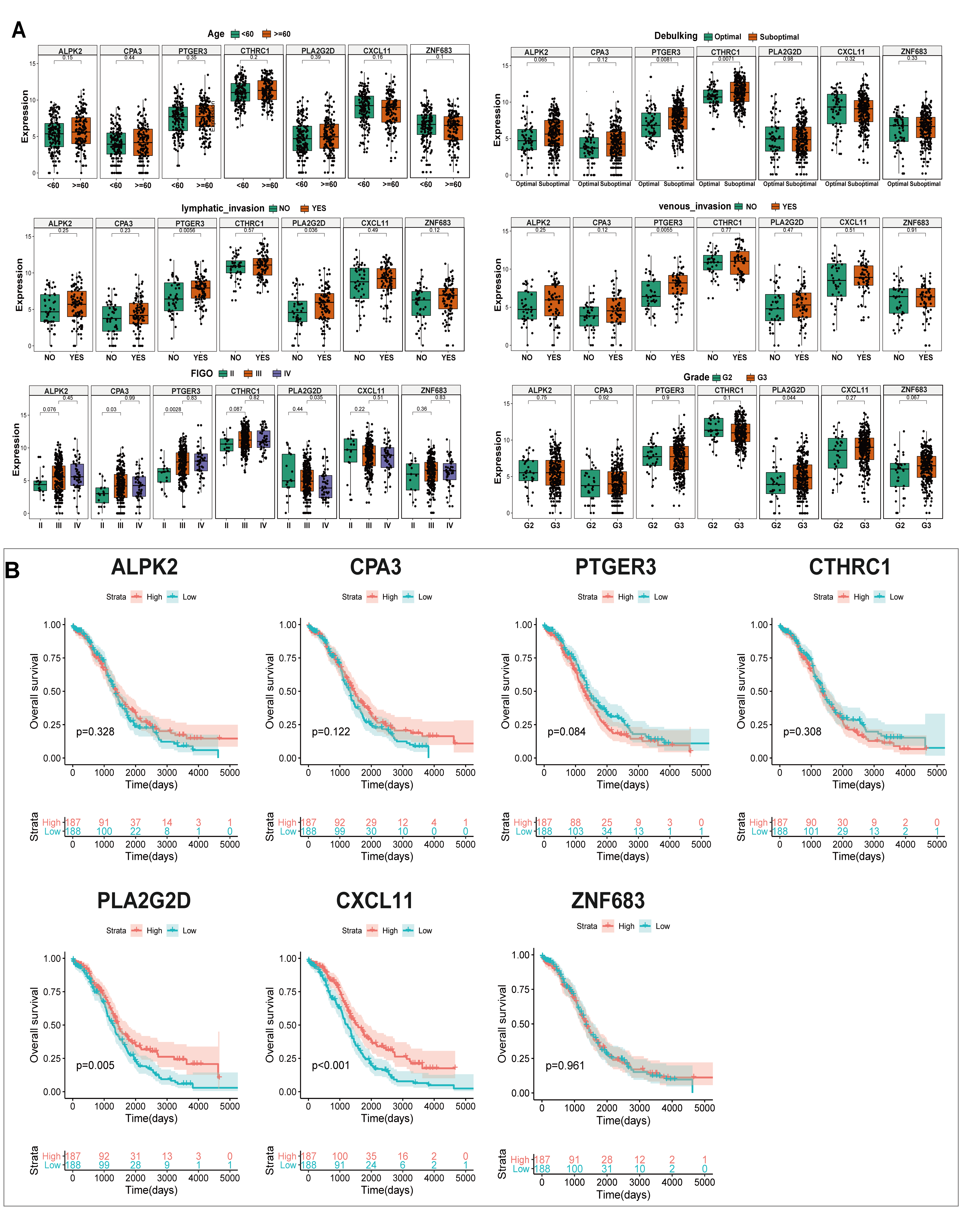

Supplement: Supplementary Figure S3 — (A) Differential analysis for the expression of seven TIME regulators in different ages; in debulking; in lymphatic and venous invasion; among FIGO stages; and in pathological grade. (B) Kaplan-Meier curve analysis of seven TIME regulators with OS in OC patients of TCGA cohorts. [file Image_3.tif]

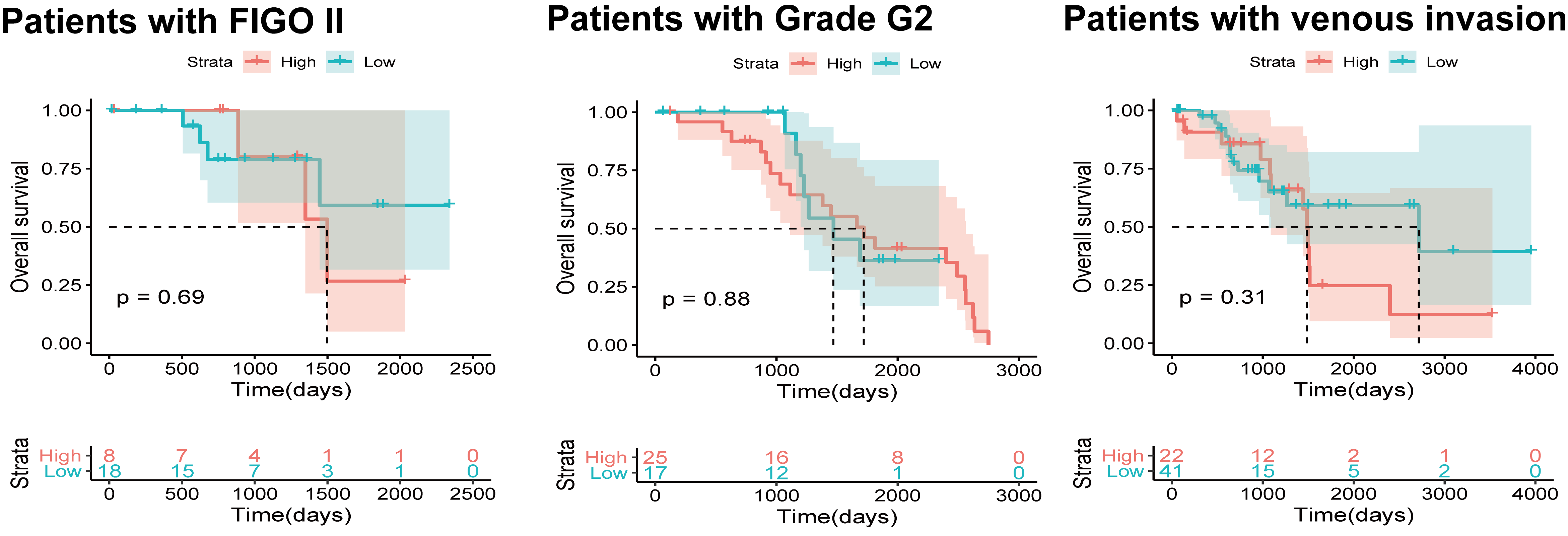

Supplement: Supplementary Figure S4 — (A) The differences of GO enrichment analysis between high TIMErisk and low TIMErisk cohorts. (B) The differences of KEGG pathway enrichment score between high TIMErisk and low TIMErisk cohorts. [file Image_4.tif]

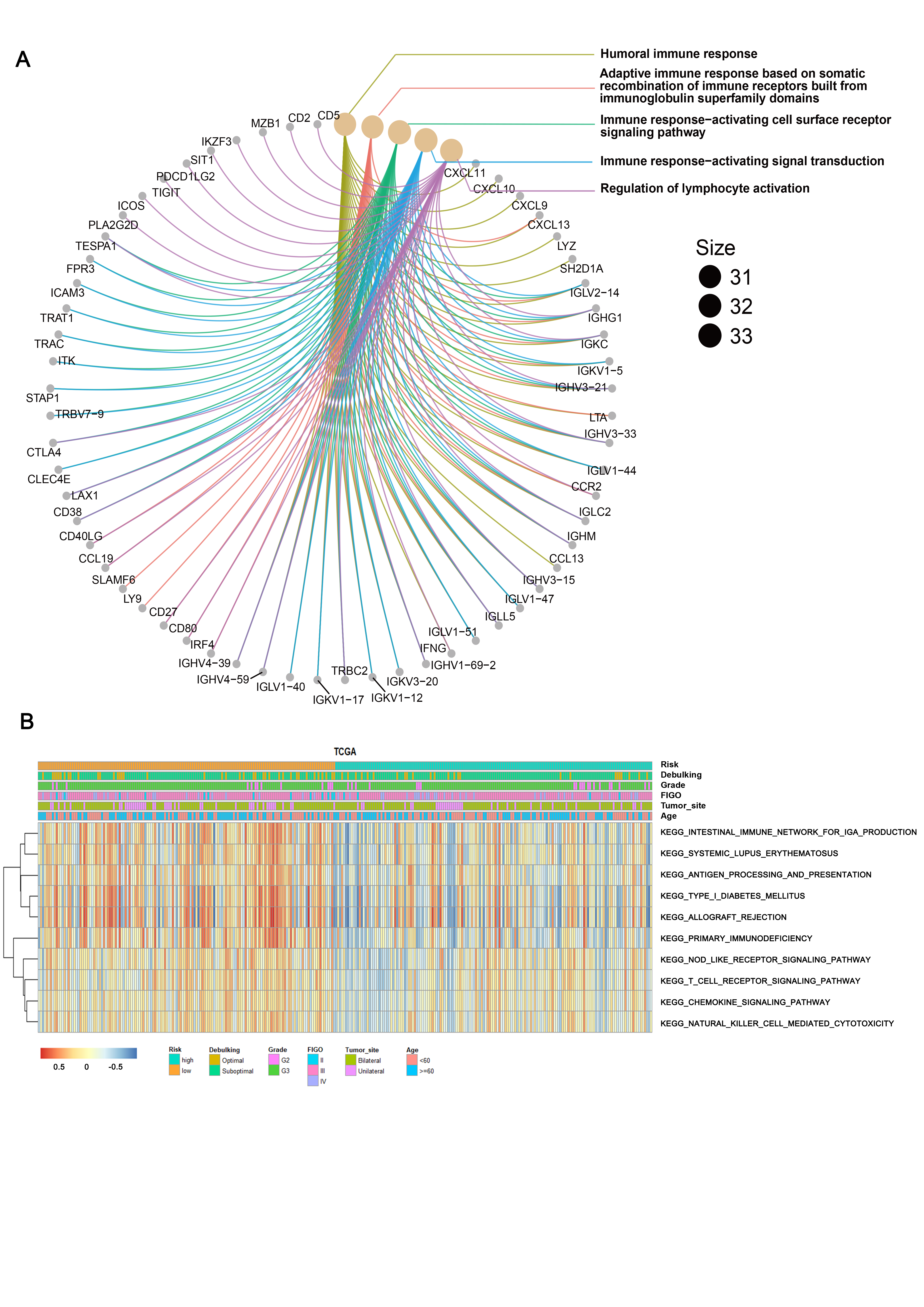

Supplement: Supplementary file 10 [file Image_5.tif]
